# Sustained Release of Triamcinolone Acetonide from an Intratympanically Applied Hydrogel Designed for the Delivery of High Glucocorticoid Doses

**DOI:** 10.1159/000358165

**Published:** 2014-04-02

**Authors:** Clemens Honeder, Elisabeth Engleder, Hanna Schöpper, Franz Gabor, Gottfried Reznicek, Jens Wagenblast, Wolfgang Gstoettner, Christoph Arnoldner

**Affiliations:** ^a^Department of Otorhinolaryngology, Medical University of Vienna, Vienna, Austria; ^b^Department of Pharmaceutical Technology and Biopharmaceutics, University of Vienna, Vienna, Austria; ^c^Department of Pharmacognosy, University of Vienna, Vienna, Austria; ^d^Department of Pathobiology, Institute of Anatomy, Histology and Embryology, University of Veterinary Medicine Vienna, Vienna, Austria; ^e^Department of Otorhinolaryngology, Johann Wolfgang Goethe University, Frankfurt/Main, Germany

**Keywords:** Intratympanic drug delivery, Glucocorticoids, Triamcinolone acetonide, Round window membrane, Inner ear therapy, Perilymph

## Abstract

The pharmacokinetic properties and tolerability of a triamcinolone acetonide poloxamer 407 hydrogel for intratympanic application were investigated in a guinea pig model. Evaluation of in vivo release kinetics showed very high initial perilymph drug levels, with clinically relevant levels present for a minimum of 10 days. Assessment of auditory brainstem response thresholds showed a minimal, delayed and transient threshold shift, which was apparent on day 3 and resolved by day 10. No relevant histological changes of the middle and inner ear structures were noted, and hair cell counts showed no significant differences between treated and untreated ears. Thus, the triamcinolone-acetonide-loaded poloxamer 407 hydrogel is an effective vehicle for sustained high-dose inner ear glucocorticoid delivery.

## Introduction

In recent years, intratympanic (IT) glucocorticoid (GC) application has become a widely used alternative to systemic therapy for the treatment of idiopathic sudden sensorineural hearing loss, autoimmune disorders and Ménière's disease [Alles et al., 2006; Battaglia et al., 2008; Garduno-Anaya et al., 2005; Trune and Canlon, 2012]. The paradigm shift from systemic to topical therapy was accompanied by large studies and clinical guidelines supporting the use of topical GCs as equivalent first line therapy for idiopathic sudden sensorineural hearing loss [Rauch et al., 2011; Stachler et al., 2012].

Systemic therapy, which is widely used in clinical practice, is hampered by limited drug delivery to the inner ear because of the minimal blood flow to the cochlea and the blood-perilymph barrier [Nakashima et al., 2003; Yang et al., 2011]. In contrast, administration of GCs directly to the round window membrane (RWM) results in high perilymph concentrations and minimizes systemic side effects [Bird et al., 2007, 2011; Parnes et al., 1999]. However, the clinical potential of the IT application of aqueous solutions is limited, since the rapid loss of fluids through the eustachian tube results in a short residence time of the applied drugs. To overcome these problems, different strategies including multiple IT injections and continuous delivery via the Silverstein MicroWick or microcatheters have been evaluated in clinical trials [Battaglia et al., 2008; Garduno-Anaya et al., 2005; Herr and Marzo, 2005; Plontke et al., 2009; Xenellis et al., 2006]. Various studies demonstrated that the use of GC-containing hydrogels for IT application results in sustained drug delivery to the perilymph without the need for repeated injections or surgical procedures required for other sustained delivery strategies [Borden et al., 2011; Paulson et al., 2008; Salt et al., 2011; Wang et al., 2009]. Sustained delivery using hydrogels - in addition to reducing the invasiveness of the application procedure - has the potential to markedly reduce the steep inner ear drug gradients which were found after 1-shot applications [Plontke et al., 2008; Salt et al., 2011].

Poloxamer 407 (POX407) hydrogels are fluid at room temperature and turn into gels at body temperature. This thermoreversibility and the mucoadhesive properties of these gels [Dumortier et al., 2006] are two desirable characteristics for easy and efficient drug delivery through the tympanic membrane. The application of a POX407 hydrogel loaded with 6% dexamethasone resulted in clinically relevant perilymph drug levels in a guinea pig model for a minimum of 42 days [Piu et al., 2011]. In addition, Wang et al. [2011] showed that inner ear GC concentrations achieved depend on the solubility of the GC used.

So far, triamcinolone acetonide (TAAc), a lipophilic GC with a high receptor-binding affinity [Jaffuel et al., 2001], has shown otoprotective effects in clinical as well as in laboratory studies [Guzman et al., 2006; Kiefer et al., 2004], though it has not been applied to the RWM in a POX407 hydrogel. In addition, achievable drug concentrations and profiles or histological effects have not yet been evaluated. Similarly, the potential to increase perilymph drug concentrations by the application of high (30%/0.3 mg/μl) concentrations of GCs has not been previously studied. These research questions were addressed in the current study.

## Methods

All animal experiments were approved by the local animal welfare committee and the Austrian Federal Ministry for Science and Research (BMWF-66.009/0159-II/3b/2011). In total, 14 pigmented guinea pigs, bred in the Department of Biomedical Research and weighing between 480 and 780 g, were used in this study.

### TAAc POX407 Formulation

A 20% (w/v) POX407 hydrogel (BASF SE, Ludwigshafen, Germany) was prepared using the cold method. This method involves slowly adding POX407 to 10 mm phosphate-buffered saline at pH 7.4. Using aseptic techniques, microcrystalline TAAc (Fagron, Barsbüttel, Germany) was suspended at a concentration of 30% (0.3 mg/μl) in the POX407 solution. Samples were stored at +4°C and resuspended by vortexing directly before use.

### Anesthesia and Surgery

All surgical procedures were performed under general anesthesia, using medetomidine (0.3 mg/kg), midazolam (1 mg/kg), fentanyl (0.03 mg/kg) and ketamine (10 mg/kg). Lidocaine (4 mg/kg) was used for local anesthesia. To aid recovery, anesthesia was partially antagonized at the end of surgery by atipamezole (1 mg/kg). All animals received carprofen (4 mg/kg) and enrofloxacin (7 mg/kg) before surgery and once per day on the following 2 days or until sacrifice. Heart rate and vascular po_2_ were measured using a pulse oximeter to monitor physical condition during surgery. Body temperature was maintained at 38°C with a heating plate.

### Application of the Hydrogel

To simulate a surgical intervention and to guarantee the application of the hydrogel to the round window niche (as opposed to a simple transtympanic delivery), the bulla was opened using a retroauricular approach. To allow for the comparison with already published studies [Piu et al., 2011; Salt et al., 2011; Wang et al., 2009], 50 μl of the hydrogel, containing a total of 15 mg TAAc, were applied under visual control to the round window niche and posterior portion of the bulla using a tuberculin syringe and a 27-gauge needle. Because of the relatively large amount of hydrogel placed in the area of the round window niche, the apical turns of the cochlea were also at least partially covered by the gel. The animal was kept in a lateral position until sol/gel transition was complete and before closure of the bulla with a denture resin (Paladur, Heraeus Kulzer, Hanau, Germany). In the last step, surgical wounds were closed using 4-0 Vicryl sutures (Ethicon, Norderstedt, Germany). After the hydrogel application, the animals were allowed to recover under a heat lamp.

### Apical Perilymph Sampling Procedure

Apical perilymph sampling was performed in a manner similar to the protocol described by Salt et al. [2006]. In short, the bulla was exposed and widely opened using a ventral approach. The apex of the cochlea was rinsed with physiological saline at least 3 times, freed from the mucosa and, again, washed 3 times with saline. Then, a small amount of histoacryl tissue glue (Braun Melsungen, Melsungen, Germany) was applied to the apex of the cochlea, and a silicone cup was created around the apex (WPI Kwik-Cast, World Precision Instruments) to facilitate perilymph sampling without loss or contamination. Finally, the apex was perforated with a needle and 5 μl of perilymph were collected in 1-μl fractions, using glass microcapillaries (Blaubrand, Wertheim, Germany). After perilymph sampling, a cerebrospinal fluid (CSF) sample was obtained. For this, a skin incision was made posterior to the vertex, muscles were scraped off the occipital bone and a hole was drilled using a small diameter diamond burr. After sharp incision of the dura, blood-free CSF was collected using microcapillaries (Blaubrand, Wertheim, Germany). Finally, a plasma sample was taken via cardiac puncture prior to euthanasia.

### Quantification of TAAc

Immediately after withdrawal, 1-μl samples were diluted with 50 μl mobile phase consisting of acetonitrile/2 mm aqueous ammonium acetate (60:40) adjusted to pH 3.2 with formic acid [Cesar et al., 2011] and stored at +4°C until analysis by high-performance liquid chromatography/mass spectrometry, comprising an Ultimate RSLC 3000 series System (Thermo Fisher Scientific, Vienna, Austria) and an API 4000 Triple Quadrupole Mass Spectrometer (AB Sciex instruments, Vienna, Austria) equipped with an electrospray ionization ion source and controlled by the Analyst 1.5 software (Dionex, Vienna, Austria). The chromatographic separation of 5-µl samples was conducted by isocratic elution using an Acclaim® 120 C_18_ reversed-phase LC column (2.1 × 150 mm, 3 µm; Thermo Fisher Scientific, Vienna, Austria) at 25°C. The run time was 15 min at a flow rate of 0.5 ml/min, and the retention time of TAAc was 1.28 min. Released TAAc was selectively detected and quantified by tandem mass spectrometry fragmentation giving a quasimolecular ion at m/z 435 [M-H]+. Multiple reaction monitoring m/z 435/397 was used for calibration to yield a linear concentration range from 5 to 100 ng/ml (correlation coefficient 0.9998) with a lower limit of detection of 1 ng/ml. Each sample series was monitored by quality control samples containing certain amounts of TAAc within the range of the calibration graph. If first measurements yielded results above the linear concentration range, perilymph samples were diluted for a second time. Actual perilymph concentrations were calculated by multiplying the measured concentrations by the dilution factor.

### Auditory Brainstem Responses

Auditory brainstem responses (ABRs) were recorded in a soundproof chamber (Industrial Acoustics Company, mac-2). To generate the sound field, a DT-48 speaker (Beyerdynamic, Germany) was placed 3 cm from the tested ear, and a K2 microphone (Sennheiser, Germany) was positioned at the level of the pinna for calibration. To acoustically isolate the tested ear, the contralateral auditory canal was filled with Ohropax classic (Ohropax, Werheim, Germany) after sufficient anesthesia. A custom-made setup, including a PC system equipped with a multifunction I/O card (National Instruments) and AudiologyLab software (Otoconsult, Germany), was used for the measurement of the auditory potentials. Stimuli used included clicks and tone bursts (3 ms duration; 1 ms rise/fall) presented in the frequency range of 1–32 kHz with 1 step/octave. Sound pressure was changed in 2-dB steps for the determination of click thresholds and in 5-dB steps for the tone bursts. Signals were amplified (80 dB), band-pass filtered between 10 Hz and 10 kHz and averaged (128×). ABRs were performed in 4 animals dedicated for perilymph sampling on day 10. Thresholds were determined by the lowest stimulus intensities that evoked a response greater than 0.5 μV, and were measured before and after the application of the hydrogel, as well as on days 3 and 10 after application.

### Histology

After perilymph, CSF and plasma sampling took place, the animals were euthanized by intracardial injection of pentobarbital (600 mg/kg) and their temporal bones were rapidly dissected. Three to 4 samples/time point were dedicated to decalcification and histological evaluation after fixation in Schaffer's solution (ethanol 80% and formalin 37%, mixed 2:1). Samples which were used for the preparation of organ of Corti whole mounts (n = 1 for days 1 and 3; n = 2 for day 10) were fixed in 4% buffered paraformaldehyde for 2 days.

For classic histology, tympanic bullae were decalcified in EDTA (8%) and carefully cut thereafter. Cutting was necessary to reduce the size of the specimen and to enable embedding in paraffin perpendicularly to the symmetry axis of the modiolus of the cochlea. Using this method, the relevant structures of middle and inner ear could be demonstrated on the same section. The preparation was cut at a thickness of 4 µm and numbered serially. In depth of interest, every 5th section was stained with hematoxylin and eosin. The sections were examined and photographed under a light microscope (Scan Scope CS/GL, Aperio Digital Pathology Systems, Oxford, UK). Evaluation comprised alterations in the following regions of interest: tympanic membrane, tympanic bulla, cochlea, spiral ganglia, RWM and ossicles. Histopathological examinations were performed blinded to the assigned treatment.

To prepare the organ of Corti whole mounts, the bony capsule was carefully removed from the cochlea. The samples were stained with phalloidin-tetramethylrhodamine B isothiocyanate (phalloidin-TRITC; Sigma-Aldrich, Vienna, Austria) and Hoechst 33342 trihydrochloride trihydrate (Molecular Probes®, Invitrogen Corp., Carlsbad, Calif., USA). Each turn of the cochlea was separately embedded in Fluorsave™ reagent (Calbiochem, Darmstadt, Germany) and analyzed by confocal microscopy. Hair cells were counted in 3 randomly selected 200-μm sections of each turn. The numbers of viable hair cells counted were expressed as a percentage of their total number expected, which was determined for every 200-μm section by counting the viable hair cells plus the missing hair cells as if still existent.

### Statistics

Data are presented as means. Error bars represent SD or SEM, as indicated below the figures. Data were analyzed using IBM SPSS (version 20). Statistical tests used were 1-way ANOVA (Tukey's honestly significant difference for post hoc analysis) and the unpaired t test, as suitable for the respective data set. Results were considered statistically significant if p < 0.05.

## Results

### Pharmacokinetics of the TAAc Hydrogel

**Fig. 1:**
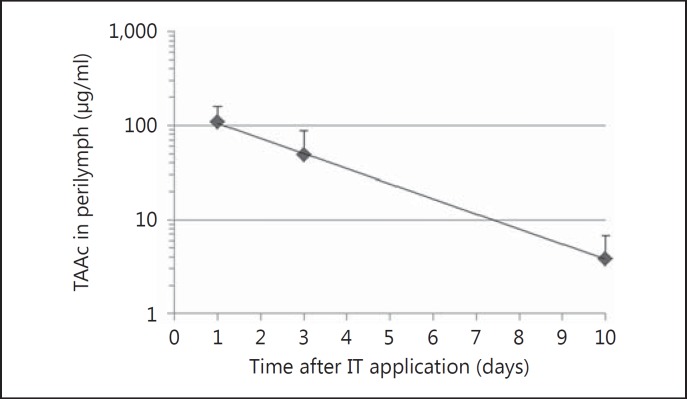
In vivo release kinetics of the 30% (0.3 mg/μl) TAAc POX407 hydrogel formulation. Perilymph levels of TAAc were measured on day 1, day 3 and day 10 after IT application of the hydrogel and averaged over the five 1-μl samples. Data are represented as means + SEM using a semilogarithmic scale (n = 3).

**Fig. 2:**
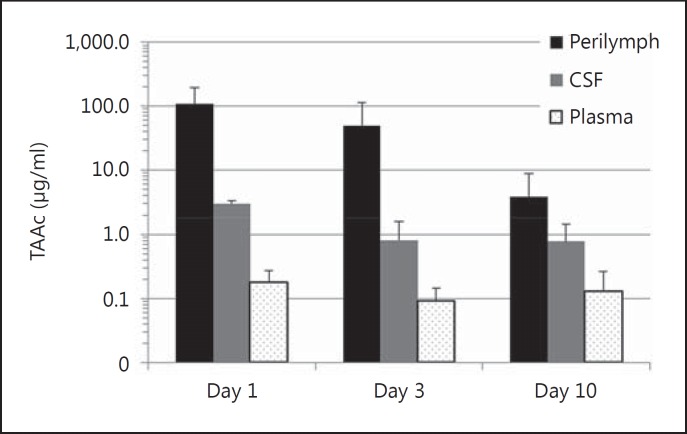
Averaged perilymph levels and CSF as well as plasma levels of TAAc, 1, 3 and 10 days after the IT application of the TAAc hydrogel. Data are presented as means + SEM using a semilogarithmic scale.

**Fig. 3:**
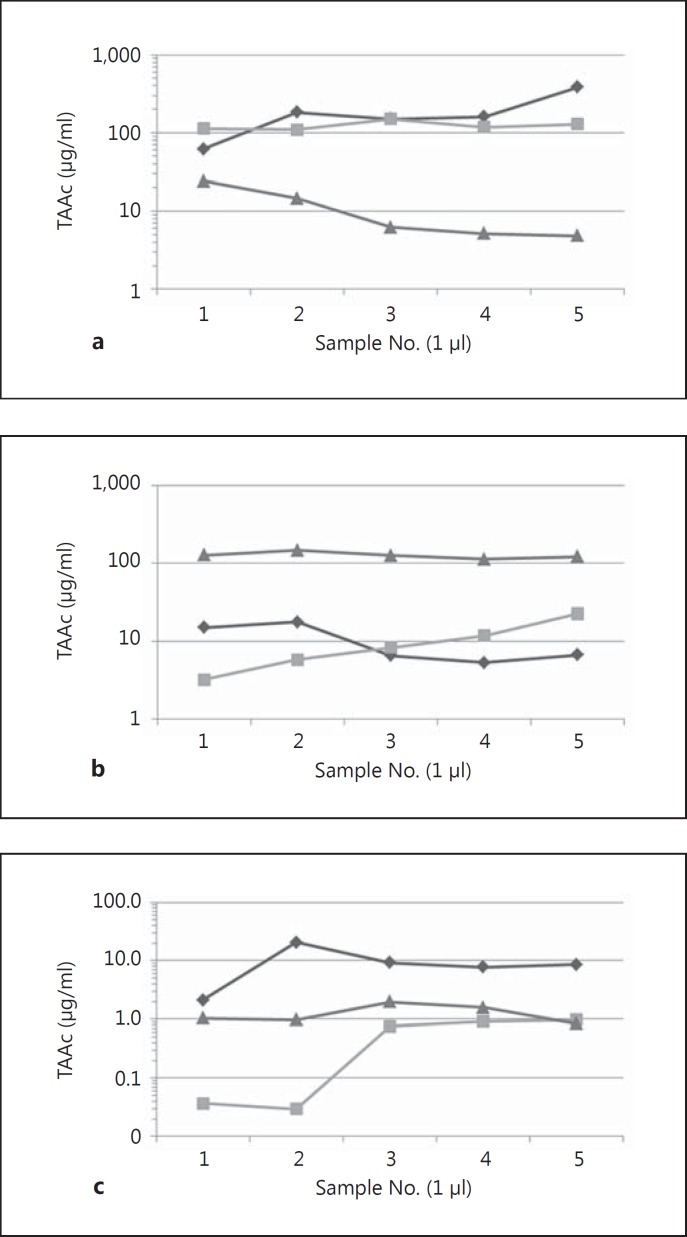
Analysis of TAAc perilymph levels in 5 consecutively sampled 1-μl fractions, 1 day (**a**), 3 days (**b**) and 10 days (**c**) after the hydrogel application. Data plotted are concentrations measured in 3 independent experiments per time point. A semilogarithmic scale is used. Note the different scale in **c**.

Guinea pigs were intratympanically injected with 50 μl of a POX407 hydrogel containing 30% (0.3 mg/μl) TAAc. Drug levels in the perilymph, CSF and plasma were monitored over a period of 10 days (recorded on days 1, 3 and 10). Initial drug levels in the perilymph, averaged over the 5 μl sampled, were 107.5 ± 51.7 μg/ml and declined to 3.8 ± 2.9 μg/ml by day 10 (fig. 1). CSF concentrations followed the same trend, with 35-fold lower TAAc levels on the first day following application (fig. 2). Plasma concentrations were relatively stable over the duration of the experiment (0.09–0.18 μg/ml), resulting in 600-fold to 30-fold lower concentrations as compared to perilymph on day 1 and day 10, respectively (fig. 2). Using this continuous round window delivery approach, the half-life of TAAc in perilymph was calculated to be 44.9 h, resulting in a stable pharmacokinetic profile. Separate analysis of 1-μl perilymph fractions did not show consistent intracochlear concentration gradients. Results ranged from basal-apical gradients to gradients pointing in the opposite direction (fig. 3).

### Auditory Brainstem Responses

**Fig. 4:**
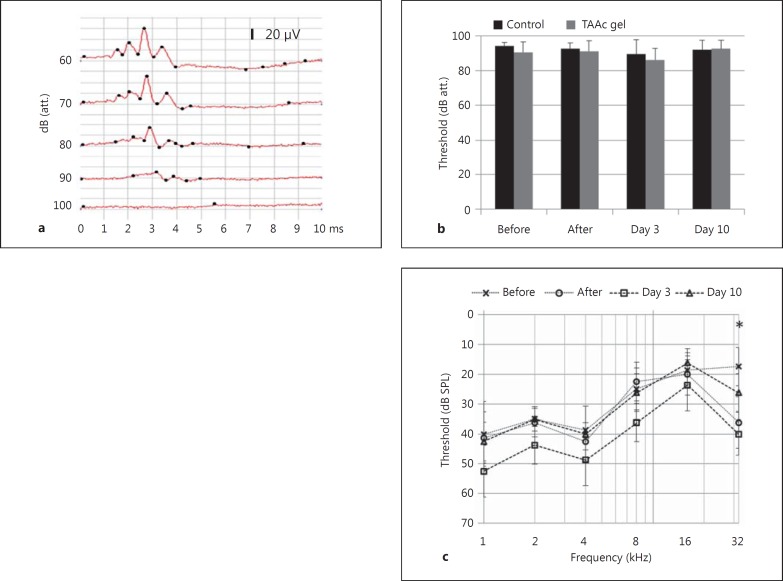
Effects of IT TAAc POX407 hydrogel on hearing thresholds. **a** Typical preoperative click ABR responses (every 5th 2-dB step plotted). **b** Click ABR thresholds of untreated control ears (n = 3) and of the TAAc hydrogel group (n = 4). Hearing thresholds were measured before and directly after the application of the hydrogel, as well as on day 3 and day 10. In the TAAc hydrogel group, click thresholds increased slightly from 91 dB (attenuation, att.) preoperatively to 86 dB (attenuation) on day 3, but fully recovered by day 10. **c** Pure-tone audiograms of the TAAc hydrogel-treated group (n = 4). On day 3, thresholds were elevated for roughly 10 dB over all frequencies, reaching statistical significance only at 32 kHz (* p < 0.05; error bars represent SD).

The application of the TAAc POX407 hydrogel led to a delayed and temporary increase in ABR click thresholds of 5 dB on day 3, which did not reach statistical significance and resolved by day 10 (fig. 4b). Frequency-specific hearing thresholds followed the same pattern, with increases of approximately 10 dB over all frequencies on day 3, which reached statistical significance only at 32 kHz (p = 0.004) and resolved by day 10 (fig. 4c). Application of a control hydrogel resulted in delayed and temporary threshold shifts comparable to the shifts after TAAc application, while the surgical opening of the bulla alone did not affect hearing thresholds (data not shown).

### Histological Evaluation

Middle and inner ears were histologically evaluated after each sample taken. The assessment was focused on pathological changes in the area of the RWM and the organ of Corti.

#### Middle and Inner Ear Sections

**Fig. 5:**
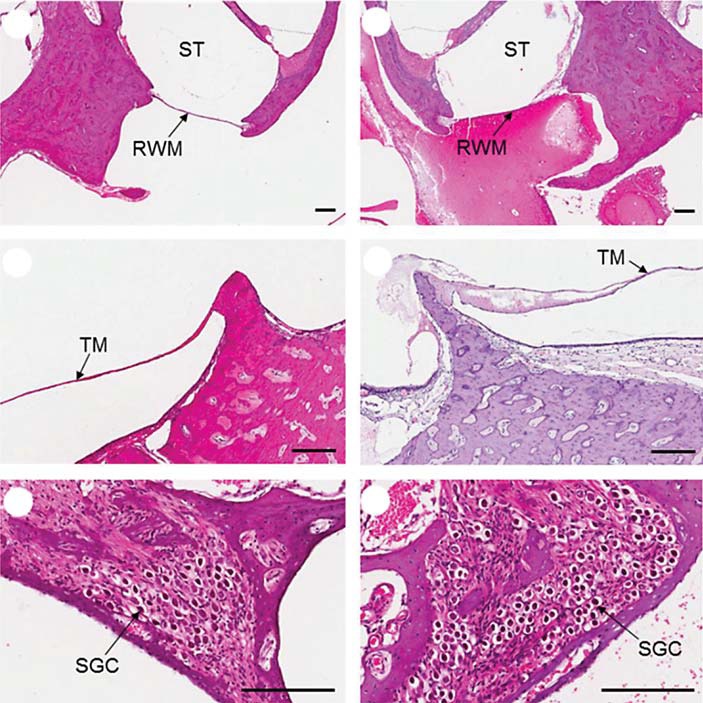
Hematoxylin-eosin-stained histological sections of decalcified guinea pig middle and inner ear samples. ST = Scala tympani; TM = tympanic membrane; SGC = spiral ganglion cells; scale bar = 200 µm. **a** RWM of a control ear without any perilymph sampling procedure or gel application. **b** RWM of an ear 10 days after TAAc treatment and perilymph sampling, with eosinophilic material covering the RWM and its niche. Blood is also visible in the scala tympani. **c** Inconspicuous tympanic membrane from an untreated ear. **d** Tympanic membrane from a treated ear 3 days after TAAc application. Blood and cell detritus coverage are apparent. **e** Spiral ganglion cells of the basal turn in a midmodiolar section in a control ear. **f** Spiral ganglion cells 3 days after TAAc treatment. Both samples show inhomogeneous and condensed nuclei irrespective of treatment.

In addition to the RWM, the tympanic membrane, spiral ganglion and ossicles were also evaluated for pathological alterations. In contrast to untreated control ears (fig. 5a), the RWMs were covered with protein-rich material in many (7/10) specimens after TAAc gel application and perilymph sampling (fig. 5b). These findings were irrespective of the sampling time point and were also found after sampling without prior hydrogel application. Nevertheless, comparison of the RWMs after TAAc hydrogel application with untreated controls did not show pathological alterations (e.g. thickening or inflammation) Tympanic membranes appeared unremarkable in controls (fig. 5c), but after TAAc treatment and perilymph sampling, they were covered with eosinophilic material in 8/10 animals, and in 5/10 animals material or blood was found inside the tympanic membranes (fig. 5d). The evaluation of the spiral ganglion cells did not show any treatment-related changes in cell size, morphology or number, but inhomogeneous nuclei were found in all groups (fig. 5e, f). Auditory ossicles appeared normal in all specimens. Additional findings include blood and some inflammatory cells in the scalae of the cochlea in almost all cases after perilymph sampling and irrespective of the prior treatment and the presence of inflammatory cells in the middle ear of 2 animals after surgery and gel application.

#### Organ of Corti Whole Mounts

**Fig. 6:**
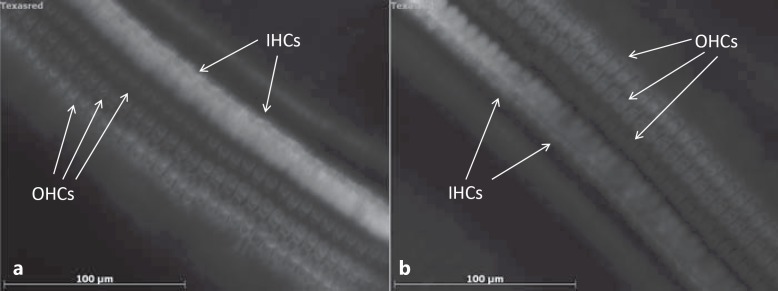
Organ of Corti whole mounts stained with phalloidin-TRITC. Three rows of outer hair cells (OHCs) and 1 row of inner hair cells (IHCs) can be identified. **a** Basal turn of an untreated control ear. **b** Basal turn 10 days after treatment with the 30% (0.3 mg/μl) TAAc hydrogel. The application of the TAAc hydrogel did not result in hair cell loss.

To evaluate a possible loss of inner and outer hair cells after TAAc application, organ of Corti whole mounts were prepared at every sampling time point. Quantitative analysis showed minor outer hair cell losses in the 3rd turn and the apex of the cochlea in all groups, but no statistically significant differences between the treated and control ears (fig. 6; table 1). Inner hair cells were completely preserved in all samples and at every time point. For statistical evaluation, samples from all time points were pooled (n = 4).

## Discussion

The data presented herein demonstrate that the IT application of TAAc in a POX407 hydrogel results in high peak perilymph concentrations, which - in the guinea pig model used - stay above 10^−6^m (0.43 μg/ml) for a minimum of 10 days. Levels above this cutoff were described to cause a GC receptor saturation and therefore to produce a maximal effect [Jaffuel et al., 2001]. IT administration was associated with a small and transient hearing threshold shift, most likely conductive in nature. Histological analysis did not identify any major histological changes of the inner or middle ear related to TAAc treatment.

In recent years, different approaches to improve IT drug delivery by the use of hydrogels, including POX407, chitosan, collagen, hyaluronic acid and poly(lactic-co-glycolic acid) nanoparticles, have been evaluated [Du et al., 2013; Endo et al., 2005; Kelly et al., 1999; Paulson et al., 2008; Tamura et al., 2005; Wang et al., 2009]. In addition to POX407, chitosan and poly(lactic-co-glycolic acid) nanoparticles have been used for the delivery of GCs, the latter two having the disadvantages of relatively short exposure times or the need for magnetic targeting for efficient drug delivery [Du et al., 2013; Paulson et al., 2008].

In comparison to the perilymph concentrations measured by apical sampling after the application of a 4.5% dexamethasone POX407 hydrogel (approx. 0.6 μg/ml) [Salt et al., 2011], the application of a 30% (0.3 mg/μl) TAAc hydrogel resulted in 26.8-fold higher peak levels (107.5 μg/ml; n-fold changes corrected for the percentage of GC hydrogel applied). This simple comparison of dexamethasone and TAAc perilymph levels corrected for the percentage of the hydrogels is a valid approach because of the similar molecular weights (MW: 392 and 434.5, respectively) and receptor binding affinities (EC_50_: 4–12 and 4–7 nm, respectively) of these two GCs [Jaffuel et al., 2001]. Interestingly, other studies addressing perilymph concentrations after the IT application of dexamethasone POX407 hydrogels, in which perilymph sampling was performed from the basal turn of the cochlea, yielded higher maximal dexamethasone concentrations. The GC levels measured after perilymph sampling from the basal turn ranged from 0.9 to 97.5 μg/ml after the application of a 1.5 and 20% dexamethasone hydrogel, respectively [Piu et al., 2011; Wang et al., 2009]. These concentrations are in the same range as the TAAc concentrations measured by our group, but higher than in a study by Salt et al. [2011], where an apical perilymph-sampling approach was used.

Very high initial GC levels can be achieved with the application of the TAAc hydrogel, and together with the long half-life of TAAc in perilymph when applied by this method (44.9 h), this results in a unique pharmacokinetic profile. The question of whether such superhigh initial GC levels, followed by a slow decrease in concentration, are superior to existent delivery protocols merits evaluation in trauma models. One day after application, CSF and plasma concentrations of TAAc were 35- and 600-fold lower than perilymph concentrations, respectively. Nevertheless, due to the very high TAAc dose used, during the 10 days of the experiment, plasma and CSF levels of TAAc stayed above 2 × 10^−7^m (0.09 μg/ ml), which causes side effects in humans [Jaffuel et al., 2001]. Given the initial TAAc perilymph concentrations, it might be a possibility to reduce the amount of GC applied in the hydrogel to minimize potential side effects.

The total amount of TAAc applied in the guinea pig model was 15 mg in 50 μl of the POX407 hydrogel so that the application of 200 μl, which has already been evaluated in a clinical trial for dexamethasone delivery [Lambert et al., 2012], would result in a total dose of 60 mg of TAAc. Due to the fact that up to 200 mg of TAAc is injected intravenously for the therapy of allergic reactions, this amount of GC appears to be safe for IT application in our eyes, especially if given in a depot formulation.

Except for a temporary ABR threshold shift on day 3, the application of the hydrogel was well tolerated. In contrast to previous findings [Salt et al., 2011; Wang et al., 2009], a hearing loss of approximately 10 dB was not evident directly after the application of the hydrogel. This can most likely be explained by the different application routes. Wang et al. [2009] as well as Salt et al. [2011] applied the hydrogel through the tympanic membrane, whereas in this study a retroauricular approach, which is also used for surgical procedures like cochlear implantation and allows for the direct visual control of the application of the hydrogel to the round window niche and posterior bulla, was used. We speculate that over time some of the hydrogel was displaced into more anterior parts of the tympanic bulla and thereby caused the conductive hearing loss evident on day 3.

Even though this change in the application procedure reduced the comparability of the hearing results, it showed that surgical opening of the middle ear prior to the application as well as postoperative alterations do not hamper effective GC delivery to the inner ear by POX407. This is important, because GCs have been successfully applied in different surgical animal models as well as clinical studies [Eastwood et al., 2010; Enticott et al., 2011; Eshraghi et al., 2007; James et al., 2008; Maini et al., 2009]. Therefore, the perioperative protection of the inner ear and the vestibular system in the setting of ear surgery could provide additional indications for the use of GC-loaded hydrogels.

Histological evaluation of the middle and inner ears, which is only of limited validity due to possible artifacts caused by the perilymph-sampling procedure, did not yield relevant pathological findings related to the TAAc hydrogel application. Since the accumulation of protein-rich material in the round window niche area was not evident after POX407 gel application without perilymph sampling in a previous study [Wang et al., 2009], this finding is most likely related to the sampling procedure. The alterations described in the tympanic membranes are most likely caused by the perilymph sampling as well, as they were not found in untreated or hydrogel-treated controls which did not undergo sampling procedures. Nevertheless, it should be mentioned that the hydrogel had contact with the tympanic membrane directly after application and that a causative effect cannot be completely ruled out. Most importantly, hair cells, spiral ganglion cells and auditory ossicles were not negatively affected by the application of the TAAc hydrogel. These findings correlate well with the unchanged hearing thresholds 10 days after the application.

Comparable to studies in humans [Bird et al., 2007, 2011], we found variable perilymph concentrations and concentration gradients after the IT application of TAAc. This could be explained by the multiple entry sites into the inner ear described for the guinea pig as an animal model, which include the RWM, the bony wall of the cochlea and the oval window [Mikulec et al., 2009; Plontke et al., 2008; Salt et al., 2012b]. Various possible entry sites in combination with the potential dislocation of the hydrogel in the middle ear could easily account for this finding, which is also supported by the presence of hydrogel remnants in anterior portions of the bulla and on apical parts of the cochlea which were found during sampling procedures. In contrast to existing publications [Plontke et al., 2008; Salt et al., 2011] we could neither find a distinguished basal-apical gradient as described for the round window delivery of GCs nor a U-shaped distribution pattern of TAAc in the perilymph as described for the dexamethasone POX407 hydrogel. These differences could possibly be explained by a longer elimination half-life of TAAc from the perilymph, which could result in a more equal distribution in the scalae of the inner ear. Another possible explanation is the longer interval between application of the drug and perilymph sampling. As the animals were allowed to recover for up to 10 days, a higher grade of variation as far as the definite hydrogel location can be expected, with regard to the entry site into the perilymph. To clarify this issue, further studies are needed - including the determination of the elimination half-time of TAAc from perilymph, as already published for dexamethasone [Salt et al., 2012a].

In addition to the favorable release kinetics of TAAc from POX407, our data also support its safety and compatibility as seen in animal models and humans [Piu et al., 2011; Singh-Joy and McLain, 2008]. This was recently complemented by safety data on the IT application of a dexamethasone POX407 hydrogel in Ménière's disease patients [Lambert et al., 2012].

In conclusion, a single shot IT application of the 30% (0.3 mg/μl) TAAc POX407 hydrogel resulted in very high initial GC levels, which were sustained over time without causing relevant side effects. These pharmacokinetic properties, together with the existing safety profile of POX407, provide a new and interesting drug delivery system that should now be tested in trauma models and could be quickly translated into clinical practice.

## Figures and Tables

**Table 1:** Inner and outer hair cell count expressed as percentage of expected hair cells

	Outer hair cells, %		Inner hair cells, %	
	TAAc gel	control	TAAc gel	control
Basal turn	99.9 ± 0.4	99.9 ± 0.4	100 ± 0	100 ± 0
2nd turn	98.1 ± 2.7	99.3 ± 2.3	100 ± 0	100 ± 0
3rd turn	95.2 ± 5.3	95.3 ± 5.6	100 ± 0	100 ± 0
Apex	94.6 ± 8.7	91.7 ± 11.7	100 ± 0	100 ± 0

Three randomly selected 200-ìm sections per turn and animal were evaluated.
